# Risk factors for refractory respiratory distress syndrome among very-low-birth-weight infants

**DOI:** 10.1186/s12887-024-05138-7

**Published:** 2024-10-24

**Authors:** Jeongmin Shin, Chang Won Choi, Byung Kook Lee

**Affiliations:** 1 Department of Pediatrics, Seoul St. Mary’s Hospital, Seoul, 06591 Republic of Korea; 2 Department of Pediatrics, Seoul National University College of Medicine, Seoul, 03080 Republic of Korea; 3 Department of Pediatrics, Seoul National University Bundang Hospital, 82 Gumi-ro 173beon-gil, Bundang-gu, Seongnam, Koreas, 13620 Republic of Korea; 4 Department of Pediatrics, Chungnam National University Sejong Hospital, Sejong, 30099 Republic of Korea

**Keywords:** Respiratory distress syndrome, Pregnancy-induced hypertension, Preeclampsia, Surfactant, Very-low-birth-weight infant

## Abstract

**Background:**

The objective was to evaluate refractory respiratory distress syndrome (RDS) risk factors among very-low-birth-weight infants (VLBWIs).

**Method:**

The data of VLBWIs born between January 2013 and December 2020 registered in the Korean Neonatal Network (KNN) were analyzed. Infants who died within 5 postnatal days or who were not given surfactant were excluded. Infants were divided into a well-responding RDS group, which received surfactant replacement therapy (SRT) only once, and a refractory RDS group, which received SRT twice or more. The associations between perinatal characteristics and refractory RDS were investigated via multivariate logistic regression analysis.

**Results:**

Multivariate logistic regression analysis revealed that low gestational age (adjusted odds ratio [aOR] = 1.26, 95% confidence interval (CI) [1.23, 1.26], male sex (aOR = 1.17, 95% CI [1.06, 1.29]), cesarean section (aOR = 1.59, 95% CI [1.38, 1.80]), maternal hypertensive disorder (aOR = 1.54, 95% CI[1.35, 1.75]), and low 5-minute Apgar scores (aOR = 1.24, 95% CI [1.12, 1.37]) were significantly associated with refractory RDS. Antenatal corticosteroid use (aOR = 0.81, 95% CI [0.73, 0.89]) and maternal chorioamnionitis (aOR = 0.79, 95% CI [0.71, 0.88]) were significantly inversely associated with refractory RDS. Compared with well-responding RDS, refractory RDS was significantly associated with increased major neonatal morbidity and mortality risk at 5 postnatal days.

**Conclusion:**

Maternal hypertensive disorder is a significant risk factor for refractory RDS. Refractory RDS was associated with unfavorable neonatal outcomes.

**Supplementary Information:**

The online version contains supplementary material available at 10.1186/s12887-024-05138-7.

## Background

Respiratory distress syndrome (RDS) and related acute lung injury (ALI) during the first several days after birth have a great impact on the subsequent clinical course of preterm infants [1]. Even with a similar gestational age (GA) or birth weight, preterm infants may have various respiratory disease courses after birth according to their perinatal characteristics [2]. Compared with infants born to mothers without such conditions, preterm infants born to mothers with intrauterine infection or inflammation often experience a favorable early respiratory course, although some of their conditions may deteriorate later [3]. Administration of antenatal steroids (ANSs) is another factor modulating the early respiratory course that induces structural and functional maturation of the lungs [4, 5]. Furthermore, sex and race are well-known modulating factors of RDS [2].

Surfactant replacement therapy (SRT) is the mainstay of RDS treatment [6]. Once surfactant is administered, it is divided into hydrophilic and hydrophobic components and forms films on the alveolar surface to prevent alveolar collapse and reduce surface tension [7, 8]. Exogenously administered surfactant is degraded by macrophages and can be recycled for the production of intrinsic pulmonary surfactant by type II pneumocytes [9]. The recycling efficacy of pulmonary surfactant is more than 90% in most mammals, including both preterm and term infants [9]. Considering the amount of surfactant used for conventional SRT and the recycling of exogenously administered surfactant, RDS should resolve after the administration of a single dose of surfactant [9]. However, in the real world, cases of refractory RDS that require multiple cycles of SRT occur. Refractory RDS may lead to ALI and the need for prolonged ventilator support. Although RDS itself is not considered a major risk factor for bronchopulmonary dysplasia (BPD) in this postsurfactant era, refractory RDS that requires prolonged ventilator care increases the risk of BPD [10]. In this regard, the identification of risk factors for refractory RDS may be crucial to guide SRT in preterm infants with RDS. If preterm infants have risk factors for refractory RDS, proactive SRT and respiratory support may reduce the risk of ALI and subsequent BPD.

In this study, we investigated the risk factors for refractory RDS requiring multiple SRT courses (≥ 2) in preterm infants with RDS using a nationwide cohort of very-low-birth-weight (VLBW) infants in Korea.

## Methods

### Study population

This study was conducted using patient data from the Korean Neonatal Network (KNN) registry, which is a prospective cohort of VLBW infants recruited from neonatal intensive care units (NICUs) across South Korea.

**Fig. 1:**
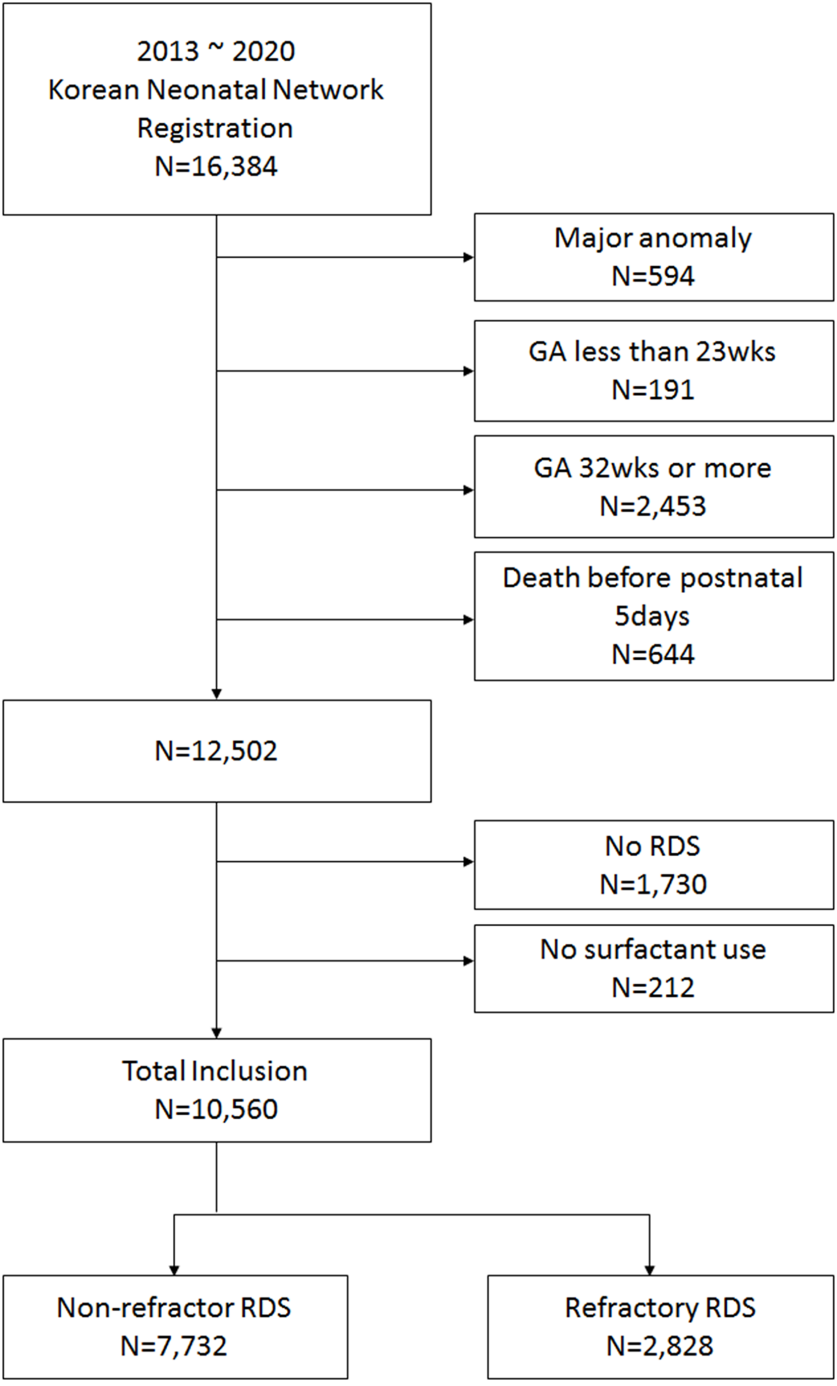
Study population Abbreviation: GA, gestational age; RDS, respiratory distress syndrome.

Among the 16,384 VLBW infants registered in the KNN who were born between January 2013 and December 2020, 191 infants born before 23 weeks GA, 2,453 infants born after 32 weeks GA, 594 infants with major congenital anomalies and 644 infants who died within 5 postnatal days, which made it difficult to evaluate their responses to SRT, were excluded. A total of 12,502 eligible infants remained. Among these infants, 1,730 infants without RDS and 212 infants who were diagnosed with RDS but were not administered surfactant were excluded. Consequently, a total of 10,560 VLBW infants composed our final study population (Fig. 1).

We divided our study population into a well-responding RDS group and a refractory RDS group. The well-responding RDS group included infants who received SRT only once for the treatment of RDS. The refractory RDS group included infants who received SRT twice or more. Among the 10,560 infants included in our study population, 7,732 were in the well-responding RDS group, and 2,828 were in the refractory RDS group.

### Definitions

Maternal chronic hypertension was defined as hypertension that developed before pregnancy or before 20 weeks GA, and preeclampsia was defined as hypertension accompanied by proteinuria and edema that developed after 20 weeks GA. These two entities share many common pathophysiologies and clinical manifestations, so we combined these two disorders into maternal hypertensive disorder, which was analyzed as a single variable. Similarly, maternal overt diabetes mellitus (DM), which referred to hyperglycemia first recognized during pregnancy but meeting nonpregnant adult standards, and gestational DM (GDM), which refers to any type of hyperglycemia first recognized during pregnancy, also share many clinical aspects, such as the delivery of large-for-gestational-age (LGA) babies, postnatal hypoglycemia, and myocardial hypertrophy caused by high insulin levels; therefore, we combined these two variables into maternal DM and analyzed it as a single variable. Completed antenatal corticosteroid (ANS) coverage was defined as cases where the administration of the last dose of ANSs was completed between 24 h and 7 days before delivery. The definitions of other variables are provided in the supplemental materials.

RDS was defined as the presence of clinically significant respiratory distress accompanied by characteristic radiologic findings consistent with RDS, such as ground-glass opacity, diffuse granular opacity, or air-bronchogram, necessitating invasive or non-invasive respiratory support.

Surfactant is a high-cost medication, and its administration by most healthcare professionals in hospitals participating in the KNN is governed by national health insurance reimbursement guidelines. According to these guidelines, prophylactic SRT is approved for a single dose administered within 2 h of birth in preterm infants with a birth weight below 1,250 g or a gestational age of less than 30 weeks. The first therapeutic SRT is indicated in cases of clinically significant respiratory distress, with characteristic radiographic and when the required fraction of inspired oxygen (FiO_2_) exceeds 40% to maintain adequate arterial oxygen tension or oxygen saturation, necessitating the use of invasive or non-invasive ventilatory support. Subsequent SRT doses are permitted after a minimum of 6 h has elapsed since the initial dose, provided that symptoms and signs of RDS persist. The dose must be administered according to the guidelines set by the national insurance system. Although there is no restriction on the number of doses in the presence of clear clinical evidence of RDS, the use of SRT for purposes such as extubation, rather than for early postnatal RDS, is not covered under national health insurance reimbursement criteria. The dosage was administered according to the specified guidelines for each medication.

### Data collection and ethical approval

The data utilized in this study were obtained from the Korean Neonatal Network (KNN) registry. The KNN was established by the Korean Society of Neonatology and the Korea Centers for Disease Control and Prevention to contribute to evidence-based disease management and to standardize and advance the care of premature infants by elucidating the characteristics and related risk factors of premature infants since 2013. As of April 2024, a total of 77 hospitals nationwide voluntarily participate in the registry. Over 80% of premature infants born in Korea weighing less than 1500 g at birth or with a gestational age of less than 32 weeks are registered in the national cohort.

This study was approved by the Institutional Review Boards of Chungnam National University Sejong Hospital (IRB No. CNUSH 2020-11-006) and Korean Neonatal Network (2021-058). Informed consent was obtained from the parents before they participated in the KNN registry and enrolled for this study. All methods were performed in accordance with the ethical standards of our institutional research committee and with the 1964 Helskinki declaration and its later amendments.

### Statistical analysis

Continuous variables are expressed as the means ± standard deviations (SDs), and categorical variables are expressed as numbers and proportions. Pearson’s χ2 test was used to compare categorical variables between groups. Student’s t test was used to compare continuous variables between groups. To adjust for confounding factors, multivariable logistic regression was performed. Confounding factors were determined based on the commonly known risk factors of RDS according to the results of previously conducted studies [11] including gestational age, male sex, and the results of univariate analysis. Adjusted odds ratios (aORs) and 95% confidence intervals (CIs) were calculated through multivariable logistic regression analysis. Factors with more than 20% missing values were excluded from the multivariable logistic regression analysis. The confounding factors used in the multivariable logistic regression for the analysis of risk factors and neonatal outcomes were GA, male sex, cesarean section delivery, in vitro fertilization (IVF), maternal DM, maternal hypertensive disorder, the presence of chorioamnionitis (CAM), complete ANS coverage and low (< 7) 5-min Apgar scores. Statistical analysis was conducted using SPSS software, version 26.0 (IBM, Corp., Armonk., NY). A *P* value < 0.05 was considered to indicate statistical significance.

## Results

### Demographic and baseline characteristics of the study population

The mean GA and birth weight of our final study population were 28^+ 1^ ± 1^+ 1^ weeks and 1,054 ± 404 g, respectively. Other demographic and clinical characteristics are presented in Table 1.

**Table 1:** Demographics and baseline characteristics of the study population

Characteristics	Total, *n* (%) (*n* = 10,560)
Gestational age (weeks)	28^+ 1^ ± 1^+ 1^
Birth weight (grams)	1,054 ± 404
Male sex	6,329 (50.6)
Cesarean section delivery	9,766 (78.1)
Maternal age (years)	33.0 ± 4.1
IVF pregnancy	3,171 (25.4)
Multiple pregnancy	4,463 (35.7)
Overt DM or GDM	1,303 (10.4)
Maternal hypertensive disorder	2,425 (19.4)
Oligohydramnios	1,576 (12.6)
Polyhydramnios	147 (1.2)
CAM	4,029 (32.8)
PPROM	4,849 (38.8)
Completed ANS administration	5,887 (47.1)

Abbreviation: IVF, in-vitro fertilization; DM, diabetes mellitus; GDM, gestational DM; CAM, chorioamnionitis; PPROM. Preterm premature rupture of membrane; ANS, antenatal steroid

### Differences in demographic and baseline characteristics between the well-responding RDS group and the refractory RDS group

Infants in the refractory RDS group were born at an earlier GA and with a lower birth weight than infants in the well-responding RDS group (28^+ 0^ ± 2^+ 1^ weeks vs. 27^+ 1^ ± 2^+ 1^ weeks, *p* < 0.001; 1,045 ± 270 g vs. 961 ± 268 g, *p* < 0.001). Infants in the refractory RDS group were more often male and delivered by cesarean Sect. (50.4% vs. 53.6%, *p* = 0.003; 71.9% vs. 83.0%, *p* < 0.001). The rates of IVF, maternal DM, and maternal hypertensive disorder were significantly greater in the refractory RDS group than in the well-responding RDS group. Complete ANS coverage was achieved less often in the refractory RDS group than in the well-responding RDS group (41.4% vs. 47.2%, *p* < 0.001). The rate of CAM was significantly greater in the well-responding RDS group than in the refractory RDS group (38.9% vs. 36.4%, *p* = 0.030).

Infants in the refractory RDS group more often had low (< 7) 5-min Apgar scores, lower blood pH and base excess in the initial blood gas analysis, and lower body temperature at the time of admission to the NICU. The time from birth to first surfactant administration was significantly shorter in the refractory RDS group than in the well-responding RDS group (34 ± 1 min vs. 52 ± 2 min, *p* < 0.001) (Table 2).

**Table 2:** Demographics and baseline characteristics of the infants in the well-responding RDS and refractory RDS groups

Characteristics	Well-responding RDS, *n* (%) (*n* = 7,732)	Refractory RDS, *n* (%) (*n* = 2,828)	*P* value
Gestational age (weeks)	28^+ 0^ ± 2^+ 1^	27^+ 1^ ± 2^+ 1^	< 0.001
Birth weight (grams)	1,045 ± 270	961 ± 268	< 0.001
Male sex	3,895 (50.4)	1,516 (53.6)	0.003
Cesarean section delivery	5,992 (71.9)	2,346 (83.0)	< 0.001
Maternal age (years)	33.2 ± 4.3	33.3 ± 4.4	0.345
IVF pregnancy	1,911 (24.7)	769 (27.2)	0.010
Multiple pregnancy	2,669 (34.5)	1,032 (36.5)	0.060
Maternal DM	839 (10.9)	261 (23.7)	0.016
Maternal hypertensive disorder	1,379 (17.8)	615 (21.7)	< 0.001
Chorioamnionitis	2,555 (38.9)	861 (36.4)	0.030
PPROM	3,027 (39.4)	1,055 (37.7)	0.101
Completed administration of ANS	3,648 (47.2)	1,172 (41.4)	< 0.001
Small for gestational age	506 (6.4)	178 (6.3)	0.891
Large for gestational age	1623 (21)	490 (17.2)	0.735
Low (< 7) 5-min apgar score	2,783 (36.2)	1,297 (46.1)	< 0.001
Initial body temperature (ºC)	36.2 ± 0.6	36.1 ± 0.7	< 0.001
Initial base excess (mEq/L)	−4.95 ± 4.00	−5.90 ± 4.47	< 0.001
Initial pH	7.27 (± 0.11)	7.26 (± 0.13)	< 0.001
Time from birth to 1st surfactant administration (min)	52 ± 2	34 ± 1	< 0.001

Abbreviation: IVF, in-vitro fertilization; DM, diabetes mellitus; PPROM, preterm premature rupture of membrane; ANS, antenatal steroid

### Risk factors for refractory RDS

Multivariate logistic regression analysis revealed that a lower GA (aOR 1.26 per 1 week decrease, 95% CI [1.23, 1.29]), male sex (aOR 1.17, 95% CI [1.06, 1.29]), cesarean section (aOR 1.58, 95% CI [1.38, 1.80]), maternal hypertensive disorder (aOR 1.54, 95% CI [1.35, 1.75]), and a low (< 7) 5-min Apgar score (aOR 1.24, 95% CI [1.12, 1.37]) were associated with an increased risk of refractory RDS, whereas CAM (aOR 0.79, 95% CI [0.77, 0.82]), and complete ANS coverage (aOR 0.81, 95% CI [0.73, 0.89]) were associated with a decreased risk of refractory RDS. IVF and maternal DM were not significantly associated with refractory RDS. Small for gestational age (SGA) and birth weight were not included in the logistic regression model because of the small number of SGA infants and the potential risk of multicollinearity with GA for birth weight. Blood pH and base deficit in the initial blood gas analysis and the time from birth to first surfactant administration were also excluded from the logistic regression analysis because of unacceptably high percentages (> 20%) of missing values (Table 3).

**Table 3:** Adjusted odds ratio of the refractory RDS group compared to the well-responding RDS group

Variables	aOR (95% CI)	*P* value
Male sex	1.17 [1.06, 1.29]	0.002
Cesarean section delivery	1.59 [1.38, 1.80]	< 0.001
Maternal hypertensive disorder	1.54 [1.35, 1.75]	< 0.001
Low (< 7) 5-min Apgar score	1.24 [1.12, 1.37]	< 0.001
Low gestational age (per 1 week decrease)	1.26 [1.23, 1.29]	< 0.001
Chorioamnionitis	0.79 [0.71, 0.88]	< 0.001
Completed administration of ANS	0.81 [0.73, 0.89]	< 0.001

Abbreviation: ANS, antenatal steroid

Adjusted by gestational age, male sex, cesarean section delivery, in vitro fertilization (IVF), maternal hypertensive disorder, the presence of chorioamnionitis (CAM), complete ANS coverage, low 5 min apgar score

### Neonatal outcomes

Neonatal morbidities were compared between the refractory RDS group and the well-responding RDS group, with adjustments for GA, male sex, cesarean section delivery, IVF, maternal DM, maternal hypertensive disorder, and low (< 7) 5-min Apgar scores. The refractory RDS group had a greater incidence of respiratory morbidities, including air leak (4.0% vs. 10.4%, aOR 1.82, 95% CI [1.42, 2.34]), pulmonary hemorrhage (4.7% vs. 11.6%, aOR 1.60, 95% CI [1.27, 2.02]), BPD and death before 36 weeks postmenstrual age (36.2% vs. 54.4%, aOR 1.20, 95% CI [1.13, 1.58]).

Infants in the refractory RDS group were more often treated for patent ductus arteriosus (PDA), either medically or surgically, than infants in the well-responding RDS group were (42.7% vs. 52.2%, aOR 1.17, 95% CI [1.17, 1.58]). Surgical treatment for PDA was performed more often in the refractory RDS group than in the well-responding RDS group (19.5% vs. 30.4%, aOR 1.17, 95% CI [1.01, 1.37]).

The refractory RDS group had a greater incidence of neurological morbidities, including high-grade (≥ 3) intraventricular hemorrhage (IVH) (18.4% vs. 32.7%, aOR 1.40, 95% CI [1.21, 1.61]), than did the well-responding RDS group. Infants in the refractory RDS group had longer durations of invasive (27.5 ± 33.8 days vs. 17.4 ± 26.7 days, *p* < 0.001]) and noninvasive mechanical ventilation (25.8 ± 25.9 days vs. 23.4 ± 21.4 days, *p* < 0.001) and hospitalization (84.0 ± 50.6 days vs. 76.7 ± 38.2 days, *p* < 0.001). The incidence of treated retinopathy of prematurity (ROP) was also higher in refractory RDS group (13.3% vs. 17.5%, *p* < 0.001). Mortality after 5 postnatal days was greater in the refractory RDS group than in the well-responding RDS group (17.9% vs. 8.5%, *p* < 0.001) (Table 4).

**Table 4:** Major neonatal outcomes between the well-responding RDS group and refractory RDS group and the adjusted odds ratios

Variables	Well-responding RDS, *n* (%) (*n* = 7,732)	Refractory RDS, *n* (%) (*n* = 2,828)	*P* value	aOR (95% CI)
Air leak	309 (4)	293 (10.4)	< 0.001	1.82 [1.42, 2.34]
Pulmonary hemorrhage	366 (4.7)	328 (11.6)	< 0.001	1.60[1.27, 2.02]
Pulmonary hypertension	611 (7.9)	514 (18.2)	< 0.001	–^*^
BPD or death before 36 weeks PMA	2,436 (31.5)	1,538 (54.4)	< 0.001	1.20 [1.31, 1.58]
Systemic corticosteroid treatment for BPD	1,087 (14.1)	575 (20.3)	< 0.001	–^*^
Treated PDA	3,301 (42.7)	1,475 (52.2)	< 0.001	1.36 [1.17, 1.58]
Surgically treated PDA	951 (19.5)	577 (30.4)	< 0.001	1.173 [1.01, 1.37]
IVH of grade 3 or higher	1,419 (18.4)	925 (32.7)	< 0.001	1.40 [1.21, 1.61]
Periventricular leukomalacia	704 (9.1)	336 (11.9)	< 0.001	–^*^
Culture proven sepsis	1,814 (23.5)	829 (29.3)	< 0.001	–^*^
NEC of stage 2 or higher	587 (7.6)	311 (11)	< 0.001	–^*^
Treated ROP	1,028 (13.3)	494 (17.5)	< 0.001	0.88 [0.75, 1.02]
Invasive ventilation duration (days)	17.4 ± 26.7	27.5 ± 33.8	< 0.001	1.00 [1.00, 1.01]
Noninvasive ventilation duration (days)	23.4 ± 21.4	25.8 ± 25.8	< 0.001	1.00 [1.00, 1.01]
O_2_ supplementation duration (days)	7.9 ± 12.2	8.9 ± 15.5	< 0.001	–^*^
Discharge with respiratory devices^¶^	1,274 (16.5)	520 (18.4)	0.021	–^*^
Admission duration (days)	76.7 ± 38.2	84.0 ± 50.6	< 0.001	–^*^
Mortality after 5 postnatal days	659 (8.5)	506 (17.9)	< 0.001	–^*^

Notes:

Adjusted by gestational age, male sex, cesarean section delivery, in vitro fertilization (IVF), maternal hypertensive disorder, the presence of chorioamnionitis (CAM), complete ANS coverage, low 5 min apgar score

^*^ These variables were not included from the final regression analysis due to insufficient model fit and small patient sample size

^¶^ Respiratory devices included home oxygen, home ventilator and home SpO2 monitoring systems

Abbreviation: BPD, bronchopulmonary dysplasia; PMA, postmenstrual age; PDA, patent ductus arteriosus; IVH, intraventricular hemorrhage; NEC, necrotizing enterocolitis; ROP, retinopathy of prematurity

## Discussion

The results of this prospective cohort study demonstrated that low GA, low birth weight, male sex, cesarean section delivery, maternal hypertensive disorder and low (< 7) 5-min Apgar scores were risk factors for refractory RDS. In contrast, CAM and complete ANS administration were protective factors against refractory RDS. Most of these risk or protective factors for refractory RDS overlap with already known risk or protective factors for RDS [12]. These findings suggest that refractory RDS shares risk factors with RDS in general. Notably, however, maternal hypertensive disorders during pregnancy were found to be a risk factor for refractory RDS. According to previous studies, maternal hypertension is regarded as a protective factor against RDS [13]. The idea of the acceleration of organ maturation caused by chronic intrauterine stress was the theoretical background of previously conducted studies [13]. In this study, we limited the study participants to VLBW infants with RDS who received SRT. In South Korea, the cost of SRT is covered by the National Health Insurance Corporation up to the third course of therapy based on the clinical decision of the physician. Beyond the third course of therapy, special documentation should be submitted to the National Health Insurance Corporation by the attending physicians. The medical and financial environment surrounding SRT enables the assumption that the number of SRT courses could be a proxy for the severity of RDS. The main pathophysiology of RDS is a deficiency of surfactant due to immature endogenous surfactant synthesis and excretion [7]. However, some preterm infants with refractory RDS require multiple surfactant administrations that exceed the size of the total physiological surfactant pool [7, 9]. This suggests that RDS is caused by a disruption of the overall process of surfactant synthesis, transportation, secretion and recycling rather than simply by surfactant deficiency [7, 14]. Another crucial pathogenic factor associated with refractory RDS may be surfactant inactivation [15]. Pulmonary surfactant can be inactivated by plasma proteins leaking into the airspace because of increased alveolar permeability. Surfactant proteins that reduce surface tension to surfactants can also be degraded by proteolytic enzymes or free oxygen radicals released by inflammatory cells recruited to the airspace [16]. Alveolar wall damage culminates in plasma protein leakage and free radical injury from the high concentration of oxygen that frequently accompanies RDS [17], especially the severe form of RDS, which may contribute to the refractoriness of SRT through the inactivation of instilled exogenous surfactant.

Maternal hypertensive disorder was identified as a risk factor for refractory RDS in this study. Furthermore, the incidence of maternal hypertensive disorder increased as the number of SRT courses increased, with the highest incidence occurring in infants who received SRT three or more times (Supplemental Table 1). Maternal hypertensive disorder, including preeclampsia, is one of the major causes of preterm delivery, along with intrauterine infection and/or inflammation [18]. The impacts of maternal hypertensive disorders on neonatal outcomes after preterm birth are not clear [19]. Immunologic and vascular dysregulation caused by maternal hypertension are thought to be major mechanisms of abnormal spiral artery development and placentation [20]. Poor placentation leads to placental insufficiency and results in fetal growth restriction (FGR) and preterm delivery [21]. Maternal hypertensive disorders can be divided into several subcategories: chronic hypertension, preeclampsia and superimposed preeclampsia [22]. These subgroups share common pathophysiologies and have similar fetal and neonatal consequences [23]. However, the interaction between maternal hypertensive disorders and neonatal RDS is not yet known. According to previous studies conducted between the 1980s and the 1990s, maternal hypertension has been thought to be associated with a reduced risk of RDS [12]. The idea of the acceleration of organ maturation caused by chronic intrauterine stress is the theoretical background of this association [12]. On the other hand, several recent studies with large cohorts have reported that maternal hypertension may increase the risk of RDS. Wen et al. [24]. reported that maternal hypertensive disorder is a risk factor for RDS requiring surfactant treatment. This study was conducted with a VLBW infant cohort registered with the Premature Baby Foundation in Taiwan. These inconsistent results on the association between maternal hypertensive disorders and neonatal respiratory morbidities might originate from changes in the obstetric care of hypertensive mothers, accessibility to SRT, progression of neonatal care and ultimately the improvement in the survival rates of preterm infants. Cohort studies conducted in the Netherlands and China have demonstrated an uncertain correlation between maternal hypertensive disorders and RDS [25, 26]. The timing and severity of maternal hypertensive disorders influence the duration of pregnancy and the timing of delivery. In studies on late preterm infants, as well as earlier studies conducted prior to the 1990s when the survival rates of preterm infants were lower and research focused on moderate preterm infants or older, maternal hypertensive disorders were often reported as a protective factor or as having no association with RDS. In contrast, more recent large-scale studies focusing on VLBWIs frequently consider maternal hypertensive disorders as a risk factor for RDS. This suggests that the impact of maternal hypertensive disorders on neonatal respiratory morbidity may vary depending on the timing of onset and severity of the condition. Therefore, large-scale studies based on pathophysiology are needed to further investigate the effects of maternal hypertensive disorders at different stages of fetal development.

Placental dysfunction or insufficiency associated with maternal hypertensive disorder has been attributed to increased maternal serum soluble fms-like tyrosine kinase-1 (sFLT-1) levels [27]. Increased sFLT-1 decreases the concentration of the circulating free vascular endothelin growth factor (VEGF) family and results in abnormal spiral artery formation in the placenta [28, 29]. Additionally, the VEGF family is known to affect pulmonary vascular development in fetuses. Pulmonary vascular development is closely related to alveolar development during lung development [30]. Preeclampsia is known to develop late in pregnancy, mainly after 34 weeks of gestation. It is known that if preeclampsia develops earlier in pregnancy or if chronic hypertension is aggravated during pregnancy, neonatal morbidities increase accordingly [31]. Different degrees and timings of exposure to low VEGF signals might partly explain inconsistent reports on the association between maternal hypertensive disorder and neonatal respiratory morbidities. It can be assumed that the longer the fetuses are exposed to a lower VEGF level, the poorer their lung maturation is [32]. Poor lung maturation may manifest as refractory RDS after birth.

It is not surprising that refractory RDS is associated with a greater incidence of various morbidities and mortality than is well-responding RDS. Respiratory morbidities and neurological morbidities such as high-grade IVH were more common in the refractory RDS group than in the well-responding RDS group. This finding suggests that the respiratory course during the first several days after preterm birth has an enormous impact on overall neonatal outcomes.

This study has strengths in that it was performed with a large prospective national cohort of VLBW infants. On the other hand, the unavailability of detailed information on the severity and timing of maternal hypertensive disorders and the lack of uniform guidelines for multiple SRT courses across centers are limitations of our study. The inability to distinguish prophylactic SRT among study population and the potential variability in the criteria of repeated SRT group are also limitations of our study that may affect the accuracy. Through this study, we were able to confirm that maternal hypertensive disorders are a risk factor for refractory RDS. However, it was not possible to determine whether maternal hypertensive disorders are a common risk factor for both RDS and refractory RDS, or an independent risk factor for refractory RDS. Further research is needed to explore whether maternal hypertensive disorders influence the severity of neonatal respiratory morbidity. Any differences in long-term neurodevelopmental outcomes between infants with refractory RDS and infants with well-responding RDS should be investigated in future studies.

## Conclusion

The clinical implication of this study is that when the risk factors for refractory RDS, including maternal hypertensive disorder, as revealed in our study, are present, clinicians should anticipate refractoriness to SRT. Proactive respiratory management, including repeated SRT courses, should be provided for preterm infants with RDS.

## Electronic supplementary material

Below is the link to the electronic supplementary material.

Supplementary Material 1

## Data Availability

This work was supported by the Research Program funded by the Korea Centers for Disease Control and Prevention. There are ethical restrictions on sharing a deidentified dataset unless permitted by the CDC of Korea. The available data were subjected to the Act on Bioethics and Safety [Law No. 1518, article 18 (Provision of Personal Information)]. Contact for sharing the data or accessing the data can be made possible only through the data committee of the Korean Neonatal Network (http://knn.or.kr) and after receiving permission from the CDC of Korea. The detailed contact information is as follows: data access committee: Yun Sil Chang (yschang@skku.edu); and ethics committee: So-Young Kim (sykimped@catholic.ac.kr).
