# Supplementary material for: Risk factors for refractory respiratory distress syndrome among very-low-birth-weight infants
Source: BMC Pediatr. 2024 Oct 24;24:677. doi: 10.1186/s12887-024-05138-7 (PMC11515632; doi:10.1186/s12887-024-05138-7)
Supplement: Supplementary file 1 — Supplementary Material 1 [file 12887_2024_5138_MOESM1_ESM.docx]

**Supplemental materials**

***Definitions of variables***

Small for gestational age: birth weight below the 3rd percentile for gestational age

Large for gestational age: birth weight beyond the 95th percentile for gestational age

Respiratory distress syndrome: Any case of respiratory distress shortly after birth, presents ground glass opacity (GGO) or other specific findings on simple radiography and shows increased oxygen demand.

Air leak: any air leak including pneumothorax, pneumomediastinum, or subcutaneous emphysema, confirmed by X-ray

Bronchopulmonary dysplasia: According to the National Institute of Child Health and Human Development (NICHD) classification, infants requiring any respiratory support, including oxygen supplementation, beyond 36 weeks postmenstrual age (PMA)

IVH grading: According to Papile’s classification^31^

NEG stage: According to modified Bell’s criteria^32^

**Supplemental table 1.** Demographics and baseline characteristics according to the number of surfactant administration.

|  | 1 Surfactant,  n (%)  (n=7,960) | 2 Surfactant,  n (%)  (n=2,234) | 3 or more Surfactant,  n (%)  (n=609) | P-value |
| --- | --- | --- | --- | --- |
| Gestational age (weeks) | 28^+0^± 2^+1^ | 27^+2^ ± 2^+1¶^ | 26^+5^± 2^+1¶ ,†^ | <0.001 |
| Birth weight (gm) | 1,047(± 227) | 978 (± 269) ^¶^ | 898(± 254) ^¶,†^ | <0.001 |
| Male | 4,007(50.3) | 1,196(53.5) ^*^ | 329(54.0) ^*^ | 0.010 |
| C/S | 6,168(77.5) ^*^ | 1,862(83.3) ^*^ | 497(81.6) ^*^ | <0.001 |
| Maternal Age | 33.2(± 4.3) | 33.3(± 4.3) | 33.6(± 4.4) | 0.154 |
| IVF | 1,952(24.5) | 611(27.4) | 160(26.3) | 0.020 |
| Multiplets | 2,737(34.4) | 840(37.6) ^*^ | 196(32.2) | 0.006 |
| GDM or Overt DM | 857(10.8) | 206(9.2) | 56(9.2) | 0.066 |
| Maternal hypertensive disorders of pregnancy | 1,427(17.9) ^*^ | 474(21.2) ^*^ | 144(23.6) ^*^ | <0.001 |
| CAM | 2,626(38.8) | 661(35.2) ^*^ | 202(40.1) | 0.011 |
| PPROM | 3,108(39.3) | 830(3.4) | 227(38.0) | 0.239 |
| Completion of ANS | 3,770 (47.4) ^*^ | 992 (41.3) ^*^ | 258(42.4) | <0.001 |
| Low 5 min AS | 2,845(35.9) | 1027(46.1) ^*^ | 276(45.6) ^*^ | <0.001 |
| Initial body temperature (℃) | 36.2(± 0.6) | 36.1(± 0.6) | 36.1(± 0.7) ^¶^ | <0.001 |
| Initial base excess | -4.93(± 4.09) | -5.81(± 4.37) ^¶^ | -6.23(± 4.82) ^¶^ | <0.001 |
| Initial pH | 7.27(± 0.11) | 7.26(± 0.13) ^¶^ | 7.26(± 0.13) | <0.001 |
| Time to 1st surfactant administration (min) | 0:52(± 2:00) | 0:36(± 1:37) ^¶^ | 0:28(± 1:14) ^¶^ | <0.001 |

Note:

* means the cell is significantly different comparing to other cells. P < 0.0083

¶ means significant difference comparing to 1 surfactant group.

† means significant difference comparing to 2 surfactant group.

Abbreviation:

C/S: Cesarean section

IVF: In-vitro fertilization

GDM: Gestational diabetes mellitus

GDM: Diabetes mellitus

CAM: Chorioamnionitis

PPROM: Preterm premature rupture of membrane

ANS: Antenatal corticosteroid

AS: Apgar score
